# Cold atmospheric plasma: Novel opportunities for tumor microenvironment targeting

**DOI:** 10.1002/cam4.5491

**Published:** 2023-02-10

**Authors:** Xiaofeng Dai, Kaiyuan Zhu

**Affiliations:** ^1^ Wuxi School of Medicine Jiangnan University Wuxi China; ^2^ Affiliated Hospital of Jiangnan University Wuxi China

**Keywords:** cancer‐associated fibroblast, cold atmospheric plasma, mesenchymal stem cell, tumor microenvironment, tumor‐associated macrophage, tumor‐infiltrating lymphocyte

## Abstract

With mounting preclinical and clinical evidences on the prominent roles of the tumor microenvironment (TME) played during carcinogenesis, the TME has been recognized and used as an important onco‐therapeutic target during the past decade. Delineating our current knowledge on TME components and their functionalities can help us recognize novel onco‐therapeutic opportunities and establish treatment modalities towards desirable anti‐cancer outcome. By identifying and focusing on primary cellular components in the TME, that is, tumor‐infiltrating lymphocytes, tumor‐associated macrophages, cancer‐associated fibroblasts and mesenchymal stem cells, we decomposed their primary functionalities during carcinogenesis, categorized current therapeutic approaches utilizing traits of these components, and forecasted possible benefits that cold atmospheric plasma, a redox modulating tool with selectivity against cancer cells, may convey by targeting the TME. Our insights may open a novel therapeutic avenue for cancer control taking advantages of redox homeostasis and immunostasis.

## Graphical abstract

CAP can possibly enhance tumor antigen secretion and enhance CD8+ TIL cytotoxicity, potentially repolarize TAMs from the M2 to the M1 state, modulate p53‐driven CAF hierarchy towards enhanced drug sensitivity, block MSCs differentiation to CAFs that is associated with reduced cancer stemness, and function as the cargo of MSCs or their derived exosomes for enhanced delivery to the tumor loci.

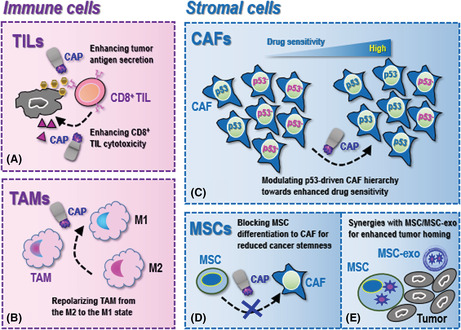

## 1 INTRODUCTION

Tumorigenesis is a complicated process not only involving genetic and epigenetic alterations of tumor cells, but also their surrounding non‐malignant cells, interactions between transformed and non‐transformed cells, as well as communications among these cellular components through the secretion of extracellular molecules. With our incremental knowledge on cancer initiation and progression, the roles of non‐transformed cells in nourishing cancer cells and cancer stemness have been recognized. The term “tumor microenvironment” (TME) has thus emerged to describe these cells and the buffering environment they foster.^1^ The TME is known to facilitate uncontrolled proliferation,^2^ accelerate tumor angiogenesis,^3^ develop cancer invasion/metastasis,^4^ promote cancer‐associated inflammation,^5^ help cancer cells escape immune surveillance,^6^ and contribute to metabolic reprogramming.^7^ With these demonstrated impacts on cancer hallmarks,^8^ the TME has been considered as the driving force and therapeutic avenue for conquering many clinical challenges such as cancer relapse and drug resistance.^9^, ^10^

Through categorizing the primary TME components and their associated onco‐therapeutic targeting modalities, we identify major TME‐modulating mechanisms that existing anti‐cancer strategies used and, accordingly, propose possible opportunities that cold atmospheric plasma (CAP) may have in the battle against cancers as an emerging TME editing tool.

## 2 PRIMARY CELLS IN TME AND THEIR ROLES IN CANCER

Primary TME components include immune cells such as tumor‐infiltrating lymphocytes (TILs) and tumor‐associated macrophages (TAMs), stromal cells such as cancer‐associated fibroblasts (CAFs) and mesenchymal stem cells (MSCs), and extracellular components such as cytokines, growth factors, hormones and extracellular matrix (ECM).

### 2.1 Immune cells in the TME

Immune cells residing in the TME include both players in the adaptive (i.e., T cells, B cells) and innate (e.g., natural killer [NK] cells, macrophages) immune responses. Here, we focus on TILs and TAMs that are dominant types of immune cells infiltrated to the TME.

#### 2.1.1 TIL

**FIGURE 1:**
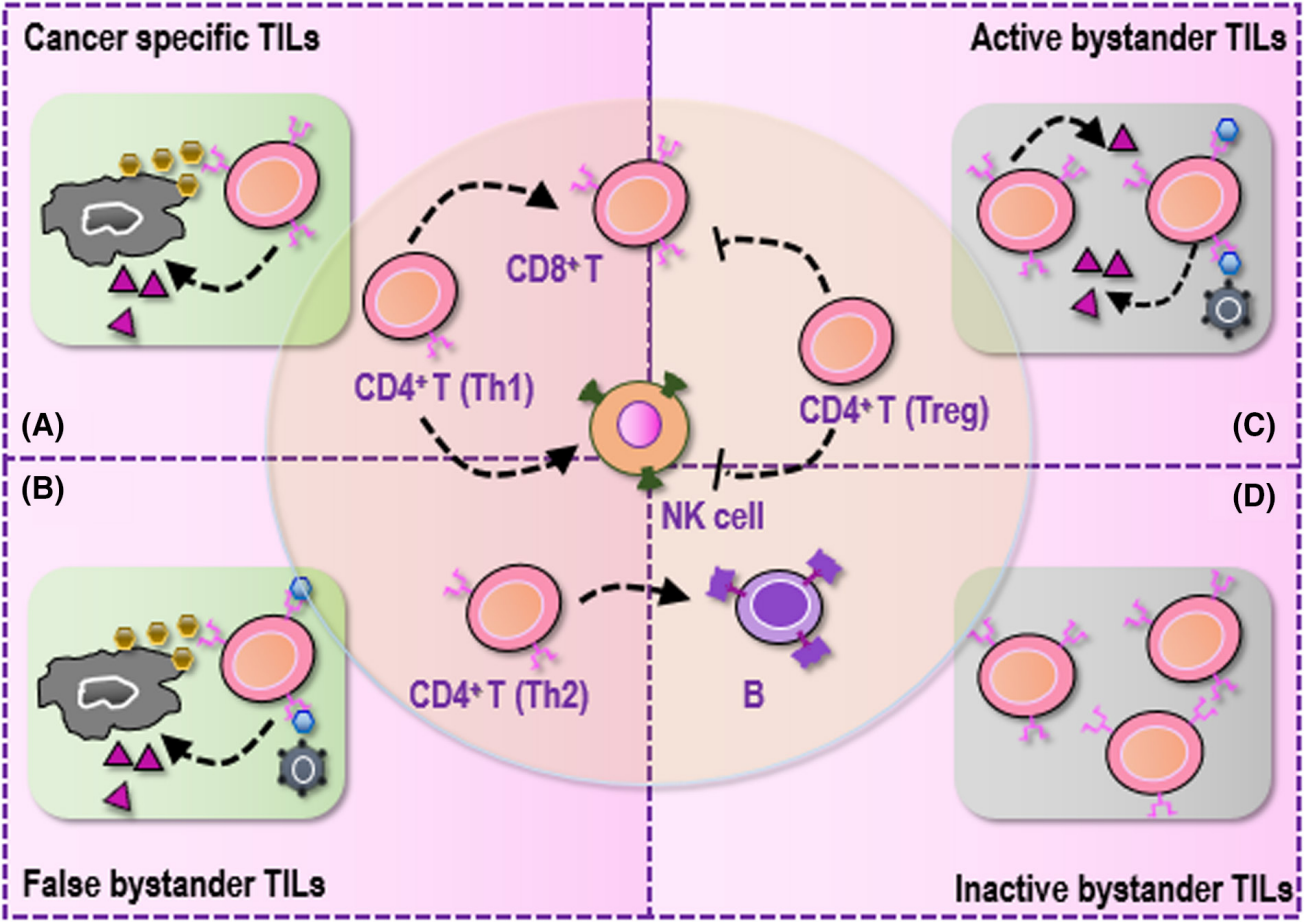
Types of tumor infiltrating lymphocytes and their primary roles in cancer. Primary tumor‐infiltrating lymphocytes (TILs) include CD8^+^ T cells, CD4^+^ T cells, B cells and natural killer (NK) cells, where CD4^+^ T cells are sub‐categorized into Th1, Th2 and Treg cells. CD8^+^ T cells are the primary TILs taking on the cytotoxicity function against cancer cells. Th1 cells and Treg cells take opposite roles, i.e., while Th1 cells activate CD8^+^ T and NK cells, Treg cells suppress them. Th2 cells activate B cells. These TILs can be categorized into four subclasses based on their contributions to anti‐cancer immunity. (A) Cancer‐specific T cells are activated in a T‐cell receptor (TCR)‐dependent way and kill tumor cells upon TCR binding of major histocompatibility complex‐presented antigens. (B) False bystander TILs recognize antigens from both cancer cells and cancer‐unrelated pathogens as they have dual TCRs and viral or bacterial antigens may also be present in tumor cells. (C) Active bystander TILs recognize tumor‐unrelated antigens in response to concurrent infection or in a TCR‐independent manner. (D) Inactive bystander TILs recognize tumor‐unrelated antigens. Both active and inactive bystander TILs do not contribute to the anti‐cancer immunity.

Tumor‐infiltrating lymphocytes, composed of CD8^+^ T cells, CD4^+^ T cells, B lymphocytes and NK cells, are lymphocytes infiltrated to the TME from the blood.^11^ CD8^+^ T cells, also known as cytotoxic T cells, are the main anti‐cancer immune cells. CD4^+^ T cells are represented by helper T cells type I (Th1), type II (Th2) and regulatory T (Treg) cells, where Th1 cells promote CD8^+^ T cell and NK cell proliferation by secreting IL2 and interferon, Th2 cells enhance the proliferation and maturation of B cells by releasing cytokines such as IL4 and IL6, and Treg cells suppress the cytotoxicity of CD8^+^ T and NK cells.^12^, ^13^ TILs can also be classified by disease specificity in the context of cancer immunity, where TILs recognizing non‐cancer peptides or being cancer ignorant are called “bystander TILs”.^14^ There is emerging evidence that bystander TILs may represent dominant TILs in the TME.^15^, ^16^, ^17^ Bystander TILs can also be sub‐grouped into “inactive bystander TILs”, “active bystander TILs”, and “false bystander TILs”, where inactive bystander TILs recognize tumor‐unrelated antigens and do not contribute to the anti‐cancer immunity, active bystander TILs recognize tumor‐unrelated antigens but are activated in response to concurrent infection or in a T‐cell receptor (TCR)‐independent manner, and false bystander TILs recognize both cancer‐specific and cancer‐unrelated targets such as viral or bacterial antigens due to the presence of dual TCRs or cross‐reactivity^14^ (Figure 1). Thus, cancer‐specific TILs and false bystander TILs are truly functional entities in the TME contributing to the anti‐cancer immunity, with most types of TILs being tumor suppressive except for Treg.

The differential roles of TILs in cancer have profound clinical implications. Sufficient tumor site infiltration of immune cells including, e.g., CD8^+^ cytotoxic T cells and CD4^+^ helper T cells, has been associated with inflamed TME that is characteristic of increased immune‐modulating chemokines,^18^ where intratumoral CD8^+^ T cell dysfunction has been proposed as a therapeutic avenue for immune‐therapies.^19^ Increased CD8^+^ cytotoxic T cells and suppressed Treg activity as triggered by curcumin was reported to be associated with halted head and neck cancer cell invasion.^20^ On the contrary, decreased CD8^+^ T cell density coupled with elevated Treg TME infiltration resulted in impaired IFNγ release from TILs and consequently a suppressive T cell contexture and accelerated colorectal cancer progression.^21^ Accordingly, low CD8^+^ T cell and high Treg density was suggested as an useful index prognostic of poor lung adenocarcinoma outcome, alone or coupled with other biomarkers.^22^ In addition, overproducing Treg‐induced cytokines generated an immune‐suppressive TME in IKKα‐deficient lung adenocarcinomas,^23^ decreasing the survival of Treg cells enhanced the anti‐tumor activity of TILs without disrupting the immune homeostasis,^24^ and suppressing Treg differentiation and infiltration was proposed as a promising approach in breast cancer immunotherapy.^25^

#### 2.1.2 TAM

Tumor‐associated macrophages, derived from monocyte TME infiltration and macrophage differentiation, are the most abundant immune cells residing in the TME. Macrophages have two main states, that is, M1 and M2. While the M1 state is tumor suppressive by releasing pro‐inflammatory cytokines such as TNF*α*, IL1, IL12, and participating in Th1 cell responses, the M2 state is tumor promotive by expressing anti‐inflammatory cytokines such as TGFβ and IL10.^26^ TAMs can be viewed as macrophages attracted at the M2 state that provide tumors with an immunosuppressive microenvironment by inhibiting T‐cell‐mediated anti‐tumor immunity.

Tumor‐associated macrophages can promote tumor progression by secreting factors such as chemokines, cytokines, proteases, and growth factors,^27^, ^28^, ^29^ and establish an immune‐suppressive TME by interplaying with Tregs.^30^ This has been demonstrated to involve many canonical cancer‐associated pathways and in varied types of tumors. Take studies in gastric cancers as an example, TAMs were shown capable of promoting cancer growth by activating the Wnt signaling,^27^ promoting tumor angiogenesis by enhancing VEGF expression,^31^ increasing cancer cell invasiveness by stimulating the NF*κ*B pathway,^32^ among the varied molecular mechanisms reported. The promotive role of TAMs has been well‐documented in other malignancies such as bladder^28^ and lung^29^ cancers for accelerated cancer cell growth, melanoma,^33^ prostate^34^ and lung^35^ carcinomas for elevated tumor‐associated angiogenesis, and ovarian,^36^, ^37^, ^38^ breast,^39^ and lung^29^, ^40^, ^41^, ^42^ cancers for enhanced metastasis.

### 2.2 Stromal cells in the TME

Stromal cells in the TME are non‐transformed cells that develop crosstalk with tumor cells and participate in tumor progression. Here we focus on CAFs and MSCs, two primary forms of TME stromal cells responsible for therapeutic hurdles such as drug resistance and cancer stemness.

#### 2.2.1 CAF

Cancer‐associated fibroblasts (CAFs), stromal cells with a mesenchymal fibroblast‐like phenotype, are originated from a variety of cells such as normal fibroblasts, CSCs, bone marrow‐derived cells, and epithelial cells undergoing the epithelial‐mesenchymal transition (EMT) process.^1^ They represent the most abundant stromal cells in the TME that accounts for approximately 50% cells in a tumor tissue.^43^ CAFs are inducted from their normal tissue‐resident fibroblasts or non‐fibroblastic mesenchymal elements by tumor cells via varied molecular mechanisms including, for example, direct contact between cancer cells and fibroblasts via Notch signaling, JAK–STAT signaling, inflammatory signaling as mediated via pro‐inflammatory cytokines (such as TNF*α*, IL1, IL6), TGFβ family ligands, RTK ligands such as FGF and PDGF, physical or chemical ECM alterations, DNA damages triggered by chemo‐ or radio‐therapies, stresses as imposed by metabolic or redox alterations, fibroblast stretching, epigenetic alterations such as histone acetylation, and SRF‐ or YAP1‐dependent transcriptional programs.^44^, ^45^ The diversified original and inductive modes of CAFs foster their heterogeneous nature, as exemplified by the existence of at least three CAP sub‐cohorts, that is, inflammatory CAFs (iCAFs), myofibroblastic CAFs (myCAFs) and antigen‐presenting CAFs (apCAFs).^46^, ^47^, ^48^ Given the aforementioned complexity of CAF, the concept of stromagenesis emerges that refers to a dynamic pro‐tumorigenesis stromal ECM editing process comprised of varied bi‐directional stromal fibroblastic crosstalks through the secretion of a variety of cytokines and metabolites in, mostly, a paracrine manner.^49^ Such a temporal–spatial heterogeneity of CAFs and the co‐evolvement of CAFs with tumor cells towards stromagenesis and tumorigenesis make CAF a critical contributor to cancer hallmarks and one possible determinant of many clinical challenges such as drug resistance, and thereby been considered as a critical roadblock in solid cancer therapy.^43^ Accumulated evidence has suggested the roles of CAFs in developing solid tumor therapeutic resistance. For example, CAFs were intrinsically resistant to gemcitabine, a standard of care for pancreatic cancer patients, and capable of secreting exosomes accelerating such a chemo‐resistance on gemcitabine exposure.^50^ A CD10^+^GPR77^+^ CAF cohort defined a chemo‐resistant lung cancer population due to persistent NFkB activation.^51^ Suppressed CAF proliferation reduced the resistance of pancreatic ductal adenocarcinomas to oxidative stress and the growth of these tumor cells.^52^

#### 2.2.2 MSC

Mesenchymal stem cells are stromal cells capable of self‐renew and multi‐lineage differentiation. MSCs can differentiate into CAFs with compelling supportive evidences favoring their pro‐tumorigenic roles, among which maintaining cancer stemness through the secretion of a variety of regulatory factors is the most frequently reported.^53^, ^54^ Specifically, CSCs can recruit and activate cells including MSCs that, in turn, modify the stroma to establish a unique microenvironment favorable for CSC maintenance and transit cancer cells from the bulk tumor state to the CSC state through the establishment of a crosstalk with cancer cells.^55^ For instance, TGFβ‐stimulated MSCs induced EMT and a CSC phenotype by activating Notch signaling in pancreatic cancers^56^ and hepatocellular carcinomas^57^; MSCs from the TME increased cancer stemness and the metastatic phenotype of prostate cancer cells through altering the CCL5‐androgen receptor pathway,^58^ promoted the tumorigenic phenotype of glioma CSCs through activating IL6/STAT3 signaling,^59^ increased the number of CSCs in ovarian tumor cells via altering bone morphogenetic protein signaling,^60^ enhanced the stem‐like properties of gastric cancer cells by upregulating Tregs,^61^ and polarized macrophages to the M2 phenotype in gastric cancers.^62^

## 3 EXISTING ONCO‐THERAPIES TARGETING TME

### 3.1 Onco‐therapeutic strategies relying on immune cells in the TME

#### 3.1.1 Targeting immune checkpoints towards restored immune surveillance

Immune checkpoints are signals capable of suppressing the immune response through regulating the antigen recognition of TCR. Cancer cells take advantages of immune checkpoints to reduce the efficacies of cytotoxic CD8^+^ T cells in the TME and thus evade the immune surveillance for uncontrolled cancer progression. Such an immune‐suppressive TME arrests many solid tumors in the “cold” state and imposes a great challenge to immune‐therapies in treating solid tumors.

**FIGURE 2:**
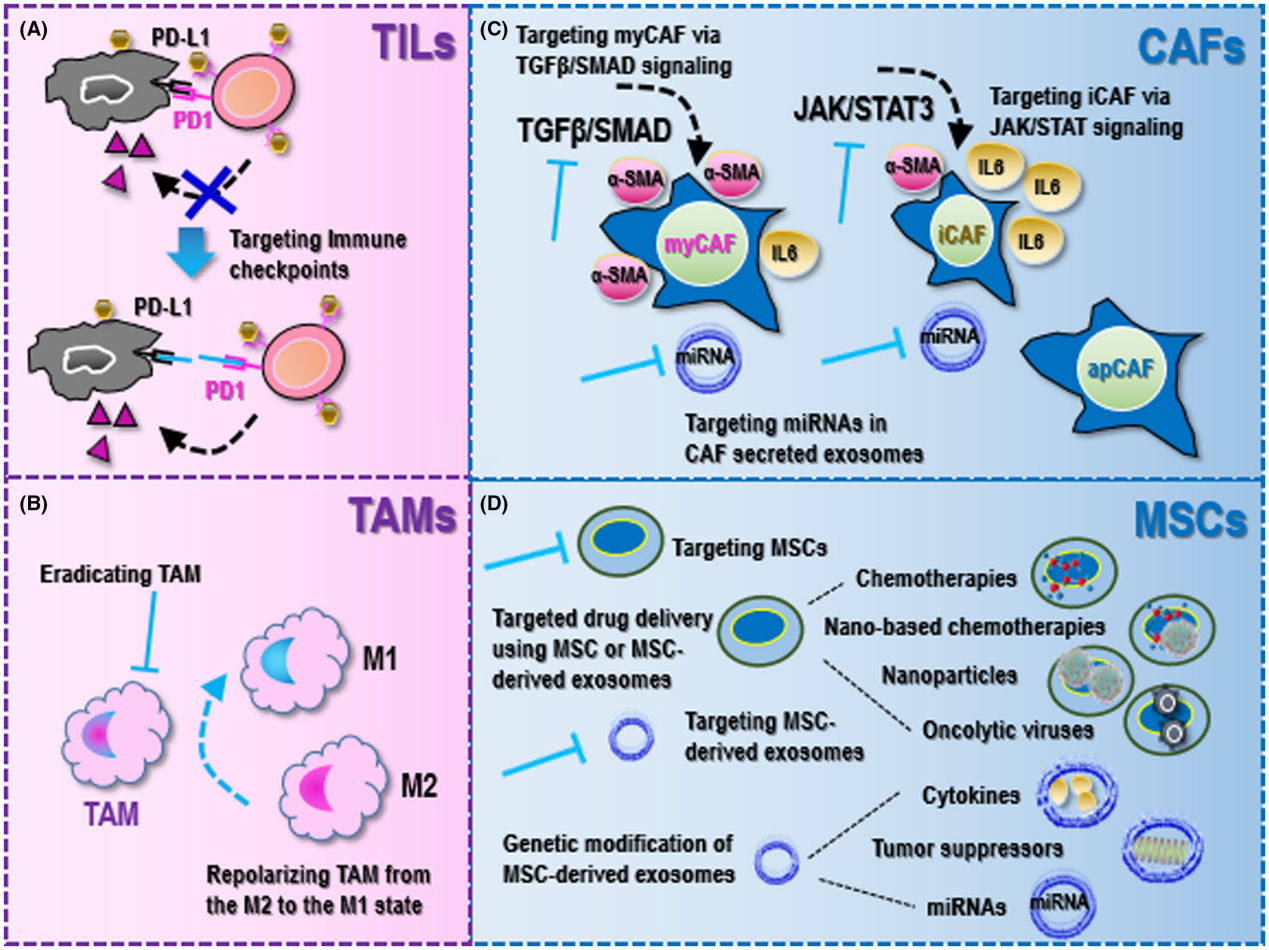
Current onco‐therapeutic strategies utilizing properties of primary tumor microenvironment (TME) cellular components. (A) Onco‐therapeutic strategies utilizing tumor‐infiltrating lymphocyte (TIL) properties largely rely on blocking immune checkpoints. (B) Onco‐therapeutic strategies targeting tumor‐associated macrophages (TAMs) either eradicate TAMs or repolarize TAMs from the M2 to the M1 state. (C) Onco‐therapeutic strategies targeting cancer‐associated fibroblasts (CAFs) mainly target myofibroblastic CAFs (myCAFs) and inflammatory CAFs (iCAFs). As myCAFs are featured by ‘high α‐SMA and low IL6’ and are activated by TGFβ/SMAD signaling, therapeutics against myCAFs are designed to target the TGFβ/SMAD axis. As iCAFs are characterized by ‘low α‐SMA and high IL6’ and are activated by JAK/STAT signaling, therapeutics killing these cells are designed to target the JAK/STAT axis. Therapeutics have also been proposed to target miRNAs in CAF‐derived exosomes. (D) Onco‐therapeutic strategies targeting mesenchymal stem cells (MSCs) can be either targeting MSCs or their derived exosomes. MSCs of different origins and their derived exosomes can be used for delivering drugs, including chemotherapies, nano‐based chemotherapies, nanoparticles, and oncolytic viruses. MSC‐derived exosomes can also be genetically modified to deliver cytokines, tumor suppressors, or miRNAs to tumors or the TME towards desirable therapeutic outcome.

Antibodies against programmed cell death 1 (PD1) and PD1 ligand (PD‐L1) have shown great promises in fighting against cancers and thus attracted much attention during recent years^61^, ^63^, ^64^ (Figure 2). PD1 is a transmembrane protein expressed on T cell surface, and CD8^+^ T cells loose cytotoxicity when PD1 binds to PD‐L1 that is expressed on the surface of cancer cells. Antibodies of PD1 and PD‐L1 allow CD8^+^ T cells to kill cancer cells by blocking interactions between PD1 and PD‐L1.^65^ Several onco‐therapeutics of this kind have been made commercially available. For instance, pembrolizumab (PD1 antibody) was shown effective in treating many types of malignancies such as triple negative breast cancers,^66^ cervical cancers,^67^ prostate cancers,^68^ gastric cancers,^69^, ^70^ esophageal cancers,^71^ gastroesophageal junction cancers,^70^ bladder cancers,^72^ pancreatic cancers,^73^ non‐small lung cancers,^74^, ^75^ melanomas,^75^ head and neck cancers,^76^ endometrial cancers,^77^ colorectal cancers,^78^ urothelial cancers^79^; and was approved by the USA Food and Drug Administration (FDA) for treating tumor mutational burden‐high solid tumors,^80^ microsatellite instability‐high solid tumors,^81^ advanced urothelial carcinomas ineligible for cisplatin‐containing chemotherapy,^82^ recurrent or metastatic head and neck squamous cell carcinomas with disease progression on or after platinum‐containing chemotherapies,^83^ recurrent locally advanced or metastatic merkel cell carcinomas,^84^ recurrent locally advanced or metastatic gastric or gastroesophageal junction adenocarcinomas expressing PD‐L1,^85^ cervical cancers expressing PD‐L1,^86^ BCG‐unresponsive non‐muscle invasive bladder cancers,^87^ metastatic non‐small cell lung cancers expressing PD‐L1 (as a first‐line therapy),^88^, ^89^, ^90^ MSI‐H/dMMR advanced unresectable or metastatic colorectal carcinomas (as a first‐line therapy),^91^ metastatic melanomas (as a second‐line therapy),^92^ and locally recurrent unresectable or metastatic triple negative breast cancers through combined use with chemotherapies.^93^ As an example of PD‐L1 antibodies, nivolumab was shown effective for treating recurrent squamous‐cell carcinomas of the head and neck,^94^, ^95^ advanced renal‐cell carcinomas,^96^ metastatic melanomas,^97^ advanced squamous‐cell non‐small cell lung cancers^98^; and was approved by FDA in the treatment of relapsed or progressive classical Hodgkin lymphomas,^99^ advanced renal cell carcinomas,^100^ metastatic non‐small cell lung cancers with progression on or after platinum‐based chemotherapies,^101^ bladder cancers,^102^ BRAF(V600) wild‐type unresectable or metastatic melanomas (as a first‐line therapy),^103^ advanced hepatocellular carcinomas,^104^, ^105^ and unresectable malignant pleural mesotheliomas when combined with lpilimumab (antibody of CTLA4).^106^

#### 3.1.2 Targeting TAM

Being an essential TME component, TAMs are tumor‐promotive. As the M2 state of TAMs is responsible for promoted tumor growth, current strategies targeting TAMs largely rely on eradicating TAMs or converting TAMs from the M2 state to the M1 state (Figure 2).

Consecutive efforts have been devoted to develop technologies targeting TAMs taking advantages of nanotechnologies. For example, desirable therapeutic outcome has been achieved in triple negative breast cancers by delivering doxorubicin, a chemotherapeutic agent, to TAMs using DOX‐AS‐M‐PLGA‐NPs (surface‐functionalized by acid‐sensitive sheddable PEGylation and modified with mannose).^107^ As another example, PLGA nanoparticles encapsulating baicalin and melanoma antigen Hgp peptide fragment 25–33 were fabricated and further loaded with CpG fragments to conjugate M2pep and α‐pep peptides on their surfaces, and the fabricated nano‐complexes were capable of transforming the M2‐like TAMs into the M1‐like phenotype.^108^ Also, the M1/M2 ratio was increased by over four folds through dual transfection of polyplexes into both tumors and TAMs in pancreatic cancer cell models.^109^ Several other approaches for TAM repolarization have also been proposed including, for example, m@Au‐D/B nanoparticle (a cancer cell membrane‐camouflaged gold nanocage loading doxorubicin and l‐buthionine sulfoximine)‐mediated photothermal therapy combined with ROS production,^110^ TAM‐targeted delivery of microRNAs with redox/pH dual‐responsive sPEG/GLC nanovectors,^111^ Ru‐based nanoparticles (Ru@ICG‐BLZ NPs),^112^ and iron chelated melanin‐like nanoparticles (Fe@PDA‐PEG).^113^

Several Chinese herb medications have also been proposed to repolarize TAMs. For instance, Astragaloside IV, a main component of nontoxic Chinese herb, was shown capable of rewiring M2 TAMs to the M1 phenotype, and thus been proposed to be combined with immune checkpoint inhibitors for colorectal cancer management.^114^ Hydrazinocurcumin repolarized TAMs to the M1 phenotype via blocking STAT3 signaling in breast cancers.^115^ Glycyrrhiza Radix et Rhizome prevented TAM M2 polarization in murine breast cancer cells via, partially, suppressing STAT6 signaling.^116^ Exosomes derived from Epigallocatechin gallate (EGCG) decreased TAM infiltration and M2 polarization in breast cancers by down‐regulating IL6 and TGFβ.^117^ Resveratrol inhibited lung cancer cell growth via suppressing STAT3‐triggered M2 polarization.^118^ HangAmDan‐B attenuated the growth of Lewis lung carcinoma (LLC) cells via inhibiting M1 polarization of TAMs.^119^ The water extract of ginseng and astragalus (WEGA) inhibited LLC cell growth by promoting M1 polarization of TAMs.^120^ PHY906, a four‐herb Chinese medicine formula (*Scutellaria baicalensis Georgi*, *Paeonia lactiflora Pall*, *Ziziphus jujuba Mill*, *Glycyrrhiza uralensis Fisch*), improved the efficacy of Sorafenib in triggering lung cancer cell apoptosis in vivo by increasing M1 TAMs.^121^

### 3.2 Onco‐therapeutic strategies relying on stroma cells in the TME

#### 3.2.1 Targeting CAF

Cancer‐associated fibroblasts are recognized players in cancer progression, with the primary contribution to carcinogenesis, among others, being therapeutic resistance. CAFs have been shown to convey resistance to radiotherapies in colorectal cancers,^24^, ^26^, ^122^, ^123^ nasopharyngeal carcinomas,^124^ and esophageal squamous cell carcinomas^125^, ^126^; to promote chemotherapeutic resistance in breast cancers,^127^ gastric cancers,^42^, ^128^, ^129^, ^130^, ^131^ head and neck cancers,^132^ pancreatic cancers,^133^ lung cancers,^134^, ^135^ bladder cancers,^136^ gastric cancers,^42^, ^128^, ^129^, ^130^, ^131^ colorectal cancers,^137^, ^138^ and ovarian cancers^139^, ^140^; to contribute to targeted therapeutic resistance in breast cancers,^141^, ^142^ prostate cancers,^143^ hepatocellular carcinomas,^144^ melanomas,^145^, ^146^, ^147^, ^148^, ^149^, ^150^, ^151^ and lung cancers^152^, ^153^; to enhance immunotherapeutic resistance in pancreatic cancers,^48^, ^154^, ^155^ lung cancers,^154^, ^156^ breast cancers,^157^ melanomas,^158^ intrahepatic cholangiocarcinomas,^159^, ^160^ urothelial cancers,^161^ esophageal cancers,^162^ and hepatocellular carcinomas.^163^

Cancer‐associated fibroblasts are heterogeneous that include myCAFs,^47^ iCAFs,^47^ and apCAFs.^48^ The myCAF cohort resides in the peri‐glandular region and is featured by high level of α‐SMA and low IL6 expression. The iCAF cells are located away from tumor cells and are characteristic of α‐SMA low and IL6 high expression. The apCAF cells are featured by the presence of major histocompatibility complex (MHC) class II (MHC II) family genes such as CD74, H2‐Aa, and H2‐Ab1 for antigen processing and presentation. While the first two subcategories of CAFs are tumor‐promotive, apCAFs play a tumor‐suppressive role. Thus, out of the three CAF forms, myCAF and iCAFs are the primary onco‐therapeutic targets.

As myCAFs are activated by TGFβ/SMAD signaling with elevated expression of α‐SMA, Ctgf, Col1α1, TAGLN, MYL9 and TPM1, therapeutic design against myCAFs largely relies on targeting TGFβ signaling (Figure 2). Galunisertib was the first oral inhibitor of TGFβ receptor with demonstrated efficacy in substantially enhancing the overall survival of unresectable pancreatic cancer patients receiving gemcitabline.^164^ M7824 was a double‐fusion protein against tumorigenesis that took action by blocking both TGFβ and PD‐L1 signalings.^165^ Several herbal medicines were reported with suppressive roles on myCAFs via blocking α‐SMA expression including, e.g., docosahexaenoic acid,^166^ resveratrol,^167^ curcumin,^168^ and silibinin.^169^

Since iCAFs are stimulated by the JAK/STAT3 axis and are featured by up‐regulated expression of IL6, IL8, IL11, CXCL1, CXCL2, CXCL12, and LIF, current strategies killing iCAFs include targeting the JAK/STAT3 axis as well as chemokines/cytokines elevated in these CAFs (Figure 2). Ruxolitinib, an inhibitor of the JAK/STAT pathway, has been shown capable of overcoming cisplatin resistance in non‐small cell lung cancers,^170^ sensitizing pancreatic cancer cells to oncolytic vesicular stomatitis viruses when coupled with polycation,^171^ restoring the sensitivity of tamoxifen‐resistant breast cancer cells,^172^ and thus been undergoing clinical trials for the treatment of metastatic HER2‐positive breast cancers^173^ and metastatic triple negative breast cancers.^174^ Blocking IL6 signaling was shown capable of rewiring the chemotherapeutic resistance of pancreatic cancers in vivo,^175^ with a clinical trial involving 140 advanced pancreatic cancer patients being launched to examine the efficacy of tocilizumab (an IL6R inhibitor) in improving the chemotherapeutic outcome (NCT02767557). In addition, combined blockage of IL6 and PD‐L1 signalings reduced pancreatic cancer progression in vivo,^176^ with the efficacy being clinically investigated (NCT04191421). Anakinra, an IL1R antagonist, improved the overall survival of pancreatic cancers in vivo,^177^ and is now under clinical investigation (NCT02021422). IL1β blockage rewired the drug resistance of pancreatic tumors in vivo,^178^ and IL1β inhibitors are being actively examined in clinics (NCT04581343). In addition, suppressing TGFβ receptors decreased STAT3 activation in pancreatic tumors in vivo,^179^ suggestive of the crosstalk between myCAFs and iCAFs as well as the possibility of concomitantly suppressing both cell cohorts using one agent.

Emerging therapeutics have been established to target exosomal microRNAs secreted by CAFs (Figure 2). For example, CAFs suppressed gastric cancer cell ferroptosis by secreting exosomal microRNA‐522, and cancer cells developed chemo‐resistance to cisplatin and paclitaxel as a result of increased exosome secretion in response to these two drugs.^129^

#### 3.2.2 Therapeutics relying on MSC

Mesenchymal stem cells, another important component in the TME, orchestrate pro‐tumor responses by supporting CSCs and interacting with non‐malignant TME components. Accumulated evidences have indicated the contribution of MSCs to cancer progression and chemotherapy resistance by maintaining cancer stemness. For instance, MSCs enhanced the self‐renewal ability of gastric cancer cells and promoted their chemo‐resistance both in vivo and in vitro through fatty acid oxidation (FAO), suggesting the feasibility of combining FAO inhibitors with chemotherapy regimens in restoring cell drug sensitivity.^180^, ^181^ MSC‐derived exosomes prevented 5‐FU triggered gastric cancer cell apoptosis both in vivo and in vitro via calcium/calmodulin‐dependent protein kinases and Raf/MEK/ERK signaling,^182^ suggestive of a promising anti‐cancer strategy by targeting MSC‐derived exosomes coupled with conventional chemotherapies (Figure 2).

The story of applying MSCs in cancer treatment is not restricted to direct targeting. One of the earliest interactions between MSCs and cancer cells is the natural homing of MSCs to the cancer milieu.^183^ The high tropism of MSCs to tumors has enabled them to be a promising tool for delivering onco‐therapeutics such as chemotherapies, nanoparticles, and oncolytic viruses^184^ (Figure 2). For example, by delivering paclitaxel using MSCs, the proliferation capacity of multiple myeloma cells was remarkably hampered,^185^ and the tumor angiogenetic ability of acute lymphoblastic leukemia was substantially reduced in vivo.^186^ The high anti‐cancer activity of nanoparticles has once attracted lots of focus in cancer treatment that, however, suffers from low tumor‐homing efficiency. Loading nano‐based chemotherapies on MSCs showed a great promise in cancer treatment. Increased drug access to the tumor site was observed by loading nano‐docetaxel on MSCs that led to potent induction of lung cancer cell death.^187^ In agreement with this, enhanced quantum dots uptake by breast cancer cells was observed when they were loaded on MSCs,^188^ and 37‐fold increased tendency of gold nanoparticles to the tumor site was reported when delivered by MSCs.^189^ Oncolytic viruses such as herpes simplex virus (HSV), adenovirus and lentivirus have been used to deliver anti‐cancer agents. Manipulated MSCs were shown capable of delivering HSV thymidine kinase (HSV‐TK) to the tumor site and significantly reducing the size and progression of glioma in vivo,^190^ suggestive of the efficacy and safety this onco‐therapeutic approach. MSCs expressing HSV‐TK have also been shown to reinforce the therapeutic value of some agents such as fluorouracil (5‐FU) in a prostate cancer xenograft model,^191^ implicative of a potential therapeutic synergy.

In addition, MSCs can secrete exosomes that possess similar properties to the source MSCs. Since exosomes can readily fuse with and evacuate cargos into the target tumor cells, they have been considered as an ideal tool for anti‐cancer agent delivery^184^ (Figure 2). For example, through incubating MSC‐derived exosomes with Dox·HCl, the drug‐loaded exosomes (Exo‐Dox) showed higher cellular uptake and anti‐tumor efficiency in osteosarcoma cells without observable cytotoxicity to normal cells.^192^ Besides, by genetically manipulating MSCs, exosomes capable of reconstructing the TME towards an unfavorable environment for the survival of neoplastic cells can be obtained. For instance, engineered MSCs with amplified INFβ expression reduced the angiogenesis capacity of prostate cancer cells by releasing INFβ to cancer cells that suppressed VEGF expression.^193^ Similarly, MSCs with enhanced INFγ expression induced glioma cell death,^194^ and hampered the proliferation of chronic myeloid leukemia cells.^195^ Apart from cytokines, attempts have also been made to express tumor suppressor genes in MSCs. For instance, MSC‐derived exosomes over‐expressing *Pten* eliminated glioblastoma cells^196^; exosomes originated from MSCs and over‐expressing apoptin substantially reduced the metabolic activity and remarkably diminished the size of liver tumors in vivo.^197^ Lastly is the modification of microRNA contents of MSCs. For example, through co‐delivery of microRNA‐124 and microRNA‐145 to glioblastoma cells via MSC‐derived exosomes, significant reduction of cancer cells was observed due to concomitant suppression on *Sox2* and *Oct4*.^198^, ^199^

Intensive clinical efforts have been devoted to MSC‐based onco‐therapeutics, most of which focused on tissue‐derived MSCs (over 50%) followed by engineered MSCs (approximately 23%) and only 1 trial was designated to evaluate the safety and efficacy of MSC‐derived exosomes.^200^

## 4 CAP AS AN EMERGING TME EDITING TOOL

Cold atmospheric plasma is composed of varied reactive oxygen and nitrogen species (RONS) including short‐lived species such as hydroxyl radical (OH·), singlet oxygen (O), superoxide (O^2−^), and nitric oxide (NO·), and long‐lived species such as hydrogen peroxide (H_2_O_2_), ozone (O_3_), anionic (OONO^−^), and protonated (ONOOH) forms of peroxynitrite. Since the first discovery on the anti‐cancer efficacy of CAP in 2007, consecutive efforts have been devoted to investigate its onco‐therapeutic impacts in varied types of cancers with demonstrated efficacies already been proven in, for example, triple negative breast cancers,^201^ bladder cancers,^202^ prostate cancers,^203^ melanomas,^204^ and pancreatic cancers.^205^ Differential cell death events can be triggered by CAP in a dose‐dependent manner^206^ that include, for example, cell cycle arrest,^203^ autophagy,^207^ apoptosis,^201^ ferroptosis,^208^ immunogenic cell death (ICD),^209^ and necrotic cell death.^210^ It has also been proposed that CAP can modulate the immunogenic response^211^ and drug sensitivity^212^ of cancer cells, halt cancer invasion and metastasis,^202^ and rewire the metabolic reprogramming of malignant cells,^213^ among others. With accumulated evidences on the selectivity of CAP against cancers and its diversified anti‐cancer properties keep being discovered, CAP has been proposed as an emerging onco‐therapeutics^214^ capable of controlling cancer cell states.^215^

Besides these preclinical studies showing the efficacy^201^, ^202^, ^203^, ^215^, ^216^, ^217^, ^218^ and safety^219^ of CAP in cancer treatment both in vitro and in vivo, the first clinical trial using CAP as an oncotherapy had been approved by FDA on July 30, 2019, in the USA (NCT04267575). Among the 20 stage IV solid tumor patients recruited in this trial, 17 patients were still alive by the study completion on 14 April 2021, suggestive of the safety and efficacy of CAP as a novel onco‐therapeutic modality.^220^

Before introducing the roles of CAP relevant to TME immune cells, we need to firstly review the cancer immunity cycle. Cancer cells in a healthy individual can be effectively killed by the cancer immunity cycle. Specifically, neoantigens are secreted by dying cells and captured by DCs, where immunogenic signals including, e.g., proinflammatory cytokines, are also released in accompany. Then, DCs present these captured antigens on MHC class I (MHCI) and MHCII molecules to T cells, which are primed to recognize and kill malignant cells carrying cancer‐specific antigens. Activated T cells then home to tumor sites and infiltrate to the TME to recognize cancer cells and take on the cytotoxic effect, where the killing of cancer cells releases additional tumor‐associated antigens to sustain the cancer immunity cycle. In cancer patients, this cycle may fail at any step. For instance, tumor antigens may not be detected, DCs may fail in presenting these antigens to T cells, T cells may not treat cancer antigens as foreign materials and thus not activated, T cells may not properly traffic to tumors, succeed in infiltrating the TME, or take on the cell killing effect due to various suppressive factors residing in the TME such as M2 TAM and CAF.^221^ Below, we characterize the possible roles of CAP in fixing the abnormal cancer immunity cycle in cancer patients by focusing on the impact of CAP on primary components aforementioned in the TME.

### 4.1 Onco‐therapeutic opportunities of CAP relevant to TME immune cells

#### 4.1.1 CAP enhances tumor antigen release and CD8 ^+^ T cell priming

**FIGURE 3:**
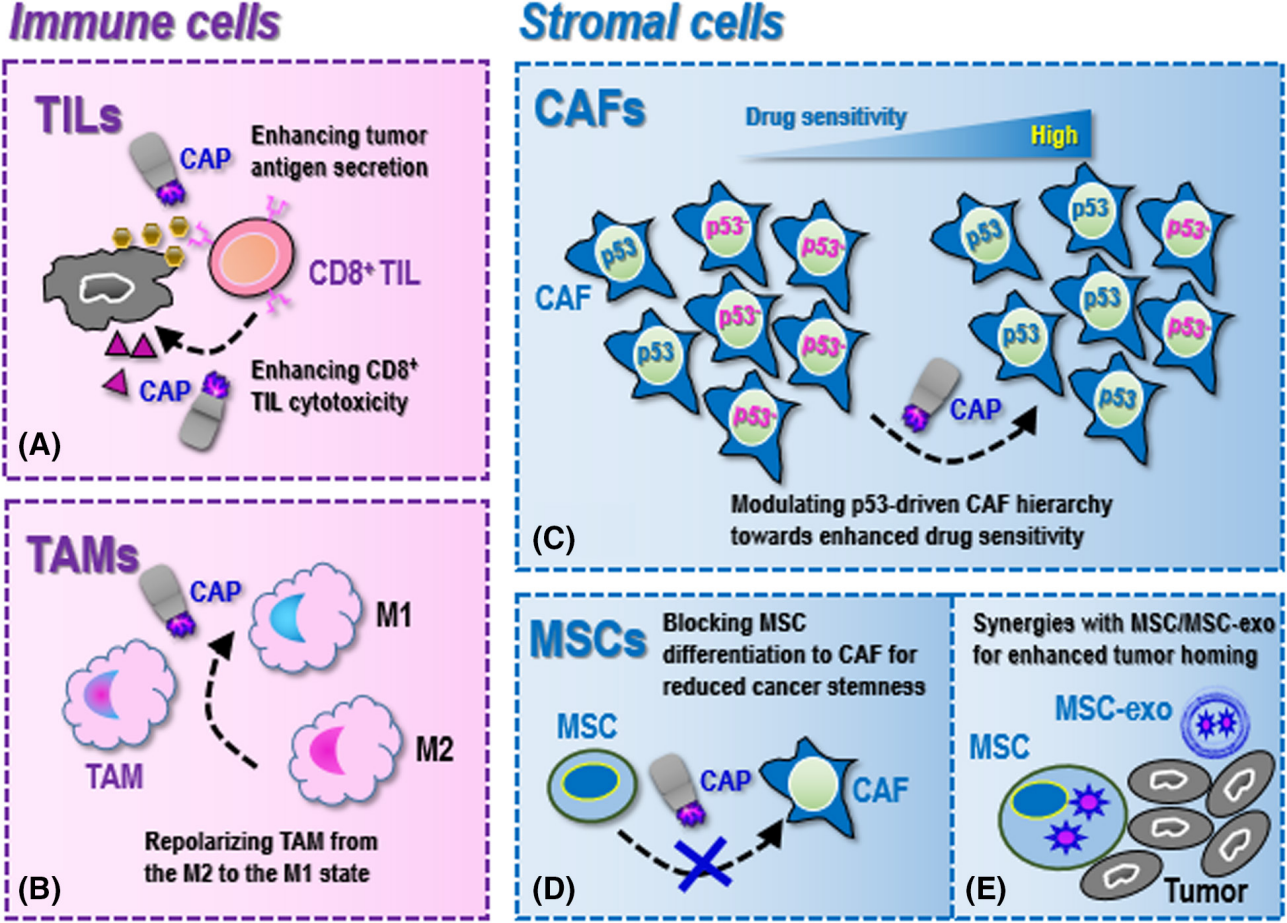
Onco‐therapeutic opportunities of cold atmospheric plasma (CAP) utilizing properties of primary tumor microenvironment (TME) cellular components. (A) For tumor‐infiltrating lymphocytes (TILs), CAP can possibly enhance tumor antigen secretion and enhance CD8^+^ TIL cytotoxicity. (B) For tumor‐associated macrophages (TAMs), CAP can potentially repolarize TAMs from the M2 to the M1 state. (C) For cancer‐associated fibroblasts (CAFs), CAP may modulate p53‐driven CAF hierarchy towards enhanced drug sensitivity. (D) For mesenchymal stem cells (MSCs), CAP may block MSCs differentiation to CAFs that is associated with reduced cancer stemness. (E) CAP can function as the cargo of MSCs or their derived exosomes for enhanced delivery to the tumor loci, where MSCs are not necessarily originated from the TME.

The presentation of cancer antigens by MHCI is essential for CD8^+^ T cells to take on their anti‐cancer cytotoxicity, where elevated intracellular ROS production can promote antigen cross presentation.^222^ CAP is a known redox modulating tool capable of enhancing cellular ROS level, and thus is possible to sensitize CD8^+^ T cells towards improved anti‐cancer activities (Figure 3). Indeed, several studies have already reported the ability of CAP in triggering ICD that is featured by enhanced cancer cell emission of danger associated molecular patterns and CD8^+^ T cell priming.^223^

#### 4.1.2 CAP repolarizes TAM from the M2 to the M1 state

It has been long and well‐acknowledged that the M1/M2 polarization of TAMs is a dynamic process in response to multiple physical factors as exemplified by oxygen tension, and the M1 TAMs are featured by an enhanced RONS forming capacity for tumoricidal activities.^224^ CAP, by definition, is a RONS generator. Thus, it is natural to assume that CAP may function as an excellent tool for TAM repolarization toward the M2 state, the study of which deserves intensive efforts (Figure 3).

### 4.2 Onco‐therapeutic opportunities of CAP relevant to TME stromal cells

#### 4.2.1 CAP restores drug sensitivity by modulating p53‐driven CAF hierarchy

Before we can understand how CAP may restore the drug sensitivity of resistant cancer cells via modulating the TME, we should firstly be acknowledged with the role of p53 mutation on CAF functionalities. The p53‐driven CAF hierarchy of pancreatic cancer cells toward a pro‐metastatic and chemo‐resistant TME has been established.^225^ Specifically, cancer cells with a gain‐of‐function p53‐mutant educated a dominant CAF cohort for a pro‐metastatic microenvironment that delayed cancer cell response to gemcitabine/abraxane, and reprogrammed the rest CAF populations towards the acquisition of more invasive features.^225^

Cold atmospheric plasma has been demonstrated capable of activating genes involved in p53 signaling in cancer cells^226^ and modulating p53 in keratinocytes.^227^ Given the essential roles played by p53 in maintaining the therapeutic‐responsive CAF hierarchy and thus cancer cell drug sensitivity, it is plausible to assume that the demonstrated efficacy of CAP in restoring the therapeutic response of many resistant cancer cells is, at least partially, attributable to the remodeled p53‐driven CAF hierarchy in the TME (Figure 3).

#### 4.2.2 CAP blocks the differentiation of MSC to CAF

Mesenchymal stem cells can be considered as the nourishing cells of CSCs and can differentiate into CAFs that are known capable of promoting tumorigenesis.^228^ The transition of MSCs into CAFs is at least partially attributable to the active secretome in the TME that includes, for example, pro‐angiogenetic factors such as VEGF and PDGF, pro‐metastatic factors such as TGFβ, pro‐inflammatory factors such as CXCL12 and IL6, and ECM modulators such as matrix metalloproteases (MMPs).^229^, ^230^

Accumulated evidences have suggested the selectivity of CAP against triple negative breast cancers,^201^, ^216^, ^231^ where significantly reduced expression of MMP1, MT‐MMP and uPA (a critical player in the plasminogen activation system that activates MMPs and degrades most ECM proteins) in response to CAP treatment was reported,^232^ suggestive of the causal relationship between the suppressive role of CAP on MMPs and its anti‐cancer effects. Interestingly, this study also reported retarded CD44 expression,^232^ the high level of which is characteristic of CSCs, associating the blocked transition from MSCs to CAFs (as indicated by reduced MMPs) with reduced cancer stemness. In agreement with this, our previous investigations in triple negative breast cancers also embraced the suppressive role of CAP on cancer stemness.^202^ In addition, another study reported reduced expression of MMP2/9 and VEGF on CAP exposure that restored the chemo‐sensitivity of breast cancer cells,^233^ implicative of a less retarded drug response as a result of blocked differentiation from MSCs to CAFs (Figure 3).

#### 4.2.3 CAP creates synergies with MSC or MSC‐derived exosomes for enhanced tumor homing

Although having been considered as a promising onco‐therapeutic strategy, the clinical application of CAP was hindered by the limited lifespan of its short‐lived species. CAP can be prepared in the form of liquid, for example, plasma activated Ringer emulsion, and can be made as the cargo of delivery vehicles alone or mixed with, e.g., hyaluronic acid^234^ for improved stability or with, e.g., hydrogel^235^ for extended release. MSCs (not necessarily originated from the TME) and their derived exosomes may function as the ideal vehicle for CAP delivery given their excellent tumor‐homing and cargo protection properties, which is expected to concentrate CAP in the tumor milieu or the TME for improved drug utility (Figure 3). Besides, as exosomes can easily pass through the blood brain barrier, MSC‐derived exosomes may offer additional benefits by delivering CAP to the brain tissues to kill tumor cells originated from or metastasized to the brain that currently lack effective and safe cure (Figure 3). In addition, it is worthwhile to explore the potential of delivering CAP in the form of oral capsules with the aid of MSC‐derived exosomes for cancer treatment that can tolerate gastric acidity (Figure 3).

## 5 CONCLUSION

This paper delineates the functionalities of the TME in tumorigenesis by classifying their primary cellular components into “immune cells” (as represented by TILs and TAMs) and “stromal cells” (as exemplified by CAFs and MSCs), reviewing current onco‐therapeutic strategies targeting these components as well as the existing clinical endeavors. Importantly, we advocate the possible roles of CAP in modulating the TME towards an environment favorable for cancer management, and identify possible molecular mechanisms driving the demonstrated selectivity of CAP against cancer hallmarks.

Cold atmospheric plasma has been proposed as an emerging tool for TME editing given its role in modulating key indexes of the TME, that is, hypoxia, acidosis, hypo‐nutrition, and inflammation.^236^ From a complementary perspective, we focus on potential impacts of CAP on the primary cellular components in the TME here. We identify and forecast the functions of CAP in enhancing tumor antigen secretion and CD8^+^ T cell cytotoxicity, repolarizing TAM from the M2 to the M1 state, modulating p53‐driven CAF hierarchy toward enhanced drug sensitivity, and blocking the differentiation of MSCs to CAFs for reduced cancer stemness. We also propose possible synergies between CAP and MSCs (not restricted to those residing in the TME) for efficient drug delivery and tumor homing. These insights may offer additional views on what redox modulation can do to resolve tumors that calls for experimental validations and deserves future attention. We do not exclude other possible impacts of CAP on the TME that may be unveiled in the future given our incremental understandings on the cellular system and the properties of CAP.

## AUTHOR CONTRIBUTIONS

Xiaofeng Dai conceived the idea and drafted the manuscript, prepared the figures, and conducted literature searching.

## FUNDING INFORMATION

This work was supported by the National Natural Science Foundation of China (Grant No. 81972789), Fundamental Research Funds for the Central Universities (Grant No. JUSRP22011). The funding bodies played no role in the design of the study and collection, analysis, and interpretation of data and in writing the manuscript.

## CONFLICT OF INTEREST

The authors declare no conflict of interest.

## Data Availability

N/A.
